# Randomly fluctuating neural connections may implement a consolidation mechanism that explains classic memory laws

**DOI:** 10.1038/s41598-022-17639-5

**Published:** 2022-08-04

**Authors:** Jaap M. J. Murre

**Affiliations:** Brain and Cognition Unit, Psychology Department, University of Amsterdam, P.O. Box 15915, 1001 NK Amsterdam, The Netherlands

**Keywords:** Human behaviour, Cognitive neuroscience

## Abstract

How can we reconcile the massive fluctuations in neural connections with a stable long-term memory? Two-photon microscopy studies have revealed that large portions of neural connections (spines, synapses) are unexpectedly active, changing unpredictably over time. This appears to invalidate the main assumption underlying the majority of memory models in cognitive neuroscience, which rely on stable connections that retain information over time. Here, we show that such random fluctuations may in fact implement a type of memory consolidation mechanism with a stable very long-term memory that offers novel explanations for several classic memory ‘laws’, namely Jost’s Law (1897: superiority of spaced learning) and Ribot’s Law (1881: loss of recent memories in retrograde amnesia), for which a common neural basis has been postulated but not established, as well as other general ‘laws’ of learning and forgetting. We show how these phenomena emerge naturally from massively fluctuating neural connections.

## Introduction

The strengths of individual neural connections in the neural networks of the brain determine our memory and knowledge. Such a connection typically consists of a synapse contacting a dendritic spine, where synapse volume, spine volume and electric connection strength tend to be correlated^1^. In the past decade, two-photon microscopy has enabled researchers to follow in an unprecedented manner the development of neural connections over minutes and days both in vitro and in vivo. Surprisingly, neural connections show rapid, large-scale intrinsic fluctuations in spine volume^2^ that seem random. Importantly, they are not necessarily driven by learning-induced plasticity^3^. This presents a huge problem for neural network models of learning, memory, and other forms of cognition^4^, because they have universally assumed that connections are stable over time (in the absence of learning). Indeed, random fluctuations in connections are routinely used as a way to *lesion* such models, for example, to model impaired semantic memory due to diffuse lesioning of cortex in semantic dementia^5^.

A model that assumes that the fluctuations in spine volume follow a Brownian motion with certain biologically motivated characteristics^3^ has demonstrated that stable long-term memory is possible and that forgetting in such a model conforms to a forgetting function reported by Ebbinghaus in 1885^6^. Here, we will demonstrate that the occurrence of massive random fluctuations in neural connections can also explain two other classic ‘laws’ of memory that were formulated in the nineteenth century but have so far eluded a satisfactory explanation in terms of neurobiological principles: very-long-term gradients (i.e., many years) with Ribot’s Law^7^ and Jost’s Law^8^.

Though we should keep in mind that many factors operate on spines and synapses independently^9^, for brevity, we will here concentrate on spine size (volume) as a short-hand for the strength or efficiency of one complete neural ‘connection’, noting that spines and synapses tend to correlate in volume and measures of LTP or LTD^1^. Spine volume distributions are strongly skewed toward small spines^10^, resembling a (stretched) exponential^11^, a gamma distribution, or lognormal distribution^12^. Similar distributions are seen for connection strengths measured in other ways^13–16^. Strength and physical size are correlated with the size of spontaneous fluctuations measured over time in vivo with two-photon microscopy^17^. If we arbitrarily call ‘weak’ connections those that have spines with heads smaller than 0.1 µm^3^^10^, we obtain the following characteristics: there are a about a 63% weak connections^10^ and about a third of these is very small and highly plastic, typically emerging and disappearing within a day^18–20^. In Alzheimer’s Dementia^18^, it is primarily the weak connections that are lost, because when they are eliminated they are often not restored, which would otherwise be the case in healthy animals, even in aging animals^21^. LTD in the smallest 20% spines leads to their disappearance. LTP promotes the stabilization of small spines^22^. Large spines, which have heads larger than 0.3 µm^3^ with mushroom-shaped heads, constitute only 6% of the distribution. These neurons’ volume will eventually reach an upper bound and will not increase after that.

Important empirical findings on neural connections are summarized by the following assumptions: (i) Connections are bounded in strength^23,24^ and (ii) do not have unlimited precision in the sense that their capacity to store information is severely limited^25^. These constraints are derived directly from neurobiology and have recently been shown to also contribute towards behavioral plausibility of models that implement them^26^. Based on the empirical studies cited above, we will, furthermore, assume that: (iii) spines fluctuate randomly in size and associated connection strength, (iv) the probability of fluctuation decreases strongly with increasing strength, (v) learning affects primarily the weakest neural connections^18–20^. We will here assume that new learning mainly stabilizes newly formed connections, for which there is neurobiological evidence^27^. Plasticity in larger spines is thought to be lower^28^, approaching zero in the largest ones. Further evidence for this importance of learning through spine stabilization is a recent study^29^ that found that pre-learning (spontaneous) spine turnover predicts learning and memory performance. A genetic manipulation that enhanced pre-learning spine turnover also enhanced learning and memory performance. Thus far, studies have found spine stabilization mainly in cortical areas^27,29^, in which small spines tend to reorganize following learning. It is important to realize that the general time course of such reorganization is in the order of minutes to hours after learning^28,30^, which sets the time scale for the forgetting processes modeled here: days to years, not seconds and minutes. Finally, we will assume that (vi) vulnerability to diffuse lesioning decreases with connection strength.

**Figure 1:**
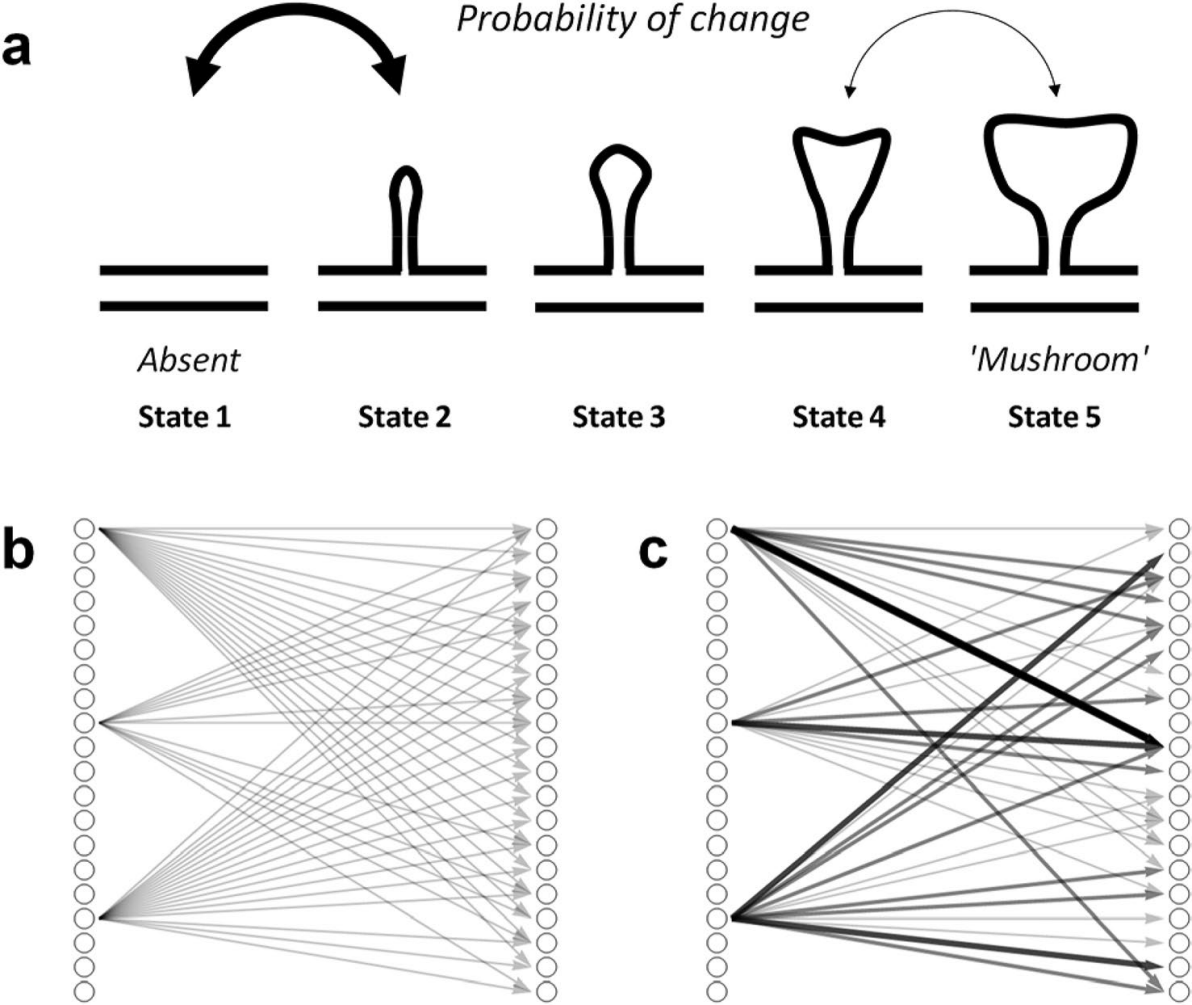
Illustration of the main principles of the model. (**a**) Example of five connection states with approximate drawings of spine shapes and two transition probabilities. (**b**) A new memory, mapping three input to twenty output neurons, initially with many weak (State 2) connections. (**c**) The same memory at a much later time: most connections have disappeared, and a few have become strong (States 4 and 5) due to random fluctuations.

The theory is implemented in a probabilistic mathematical model (illustrated in Fig. 1a), where each neural connection is in one of *S* states (assumption i-ii), numbered 1 (zero strength) to *S* (highest strength, arbitrarily set to strength 1). At each point in (discrete) time, there is a non-zero probability ${p}_{ij}$ that a connection moves from its current state *i* to a higher or lower adjacent state *j* and (iii) this probability is much smaller for higher states (iv). This results in each individual connection conforming to a random walk with reflecting boundaries^31^.

To understand its emergent behavior, we suppose a new memory is learned by connecting a memory (input) cue consisting of *A* input neurons to *B* output neurons. With the simplest learning rule, a random fraction ${p}_{i,i+1}\mu$ (with $0\le \mu \le 1$ for $\text{i = 1}$ and $\mu =0$ for $\text{i > 1})$ of the input connections to each output neuron will move from state 1 (zero state) to 2 (assumption *v*, Fig. 1b). Neurobiologically speaking, at this point the spine is turns from its fragile filopodia-like form to a more stabilized form that can survive hours or longer^30^. Activation of a large enough fraction of the input neurons would now fire the output neurons. This, in many variations, has been a standard implementation of learning in neural network models for half a century^32,33^. If we now allow the connection states to fluctuate randomly, they will eventually converge to an equilibrium distribution. In the “Methods” section below, it is proven that we can always select ${p}_{ij}$ such that the equilibrium distribution resembles empirical distributions of connection strengths^12^ and that at the same time we can select transition probabilities such that stronger connections have a lower transition probability (i.e., lower plasticity). The fluctuations will cause continued forgetting until a learned memory is eventually lost. Until that moment, the connections will retain enough information, such that when a sufficiently large portion of the original input pattern is presented, most or all of the associated output pattern can be retrieved. Moreover, the random state transitions will cause a small portion of the connections to become strong and resilient while many of the weak connections are lost (Fig. 1c). Though the memory (input–output mapping) is functionally similar, albeit weaker, its structure has undergone a change: from very plastic and vulnerable to diffuse lesioning to not very plastic and resilient to such damage. This approach to memory consolidation resembles somewhat the model presented in^34^, which is based on fluctuations of number of connections between two neurons (with multiple ‘compound’ connections between two neurons), rather than connection strength. This model, however, does not address the effects of empirical findings on fluctuations in connection size, does not reference the effects on resilience, and also does not discuss implications for the laws of learning, forgetting, and retrograde amnesia.

### Results

The theory, which I will call the Spine Drift Theory, sketched above reconciles the observed widespread fluctuations in neural connection strength with well-known characteristics of long-term memory^35,36^. Because it consists of a large population of connections, each of which has intrinsic fluctuations, a memory as a whole will follow a plausible course of forgetting^3^. Forgetting here is mainly due to fluctuations that will drive learned connection strengths toward their equilibrium distribution, which is a form of strength decay rather than interference through additional learning. There is a long-standing debate in memory psychology over whether forgetting is caused by decay or interference^37–39^. The biological processes addressed here leave no doubt that memories must also decay because their neural basis constantly shifts and erodes. Mathematical analysis of this process (see “Methods” C.5) shows that the initial portion of the forgetting curve tends towards a power curve while it tends towards an exponential curve (or plateau) for very remote times. This shape resembles a typical forgetting curve^35^, though one should bear in mind that the precise shape of forgetting is affected by many factors.

With repeated learning of the same material, fewer and fewer connections will remain in the lowest state and the effect of additional learning in this manner will be progressively smaller, leading to the characteristic negatively accelerating, exponential learning curve^40^ (see “Methods” C.4).

Ribot's Law^7^ states that with brain damage, recent memories are more vulnerable than old memories. Since its formulation in 1881, the retrograde amnesia gradient has been observed in countless studies with experimental animals and human subjects^41^. According to assumption (vi) above, old memories have more strong connections with large spines and are therefore much less vulnerable to the effects of diffuse lesioning, as was found with Alzheimer's Disease^18^. Convergence to the equilibrium distribution may take many years (see “Methods” C.3). Hence, complete loss of memories may also follow a very long time course (for certain memories), as has frequently been observed in humans with Alzheimer’s Disease^41^. Most current theories cannot explain a decades-long memory consolidation process, which greatly exceeds the known time constants of the hippocampus-cortex dialog^42^, which was among others advocated by Squire^43^. Indeed, early connectionist implementations^44–47^ of this so called ‘Standard Theory’ of memory consolidation could explain very long-term Ribot gradients only by assuming a nearly lifelong timespan for the hippocampus-to-cortex consolation process. An alternative theory, called ‘Multiple-Trace Theory’^48,49^, proposed that the hippocampus remains always involved in memory retrieval and denies the importance of a hippocampus-cortex dialog for long-term memory consolidation. Rather, a mechanism of replication of neural memory traces is seen as the main mechanism to make memories more resilient over time. The theory introduced in this paper proposes yet another mechanism.

**Figure 2:**
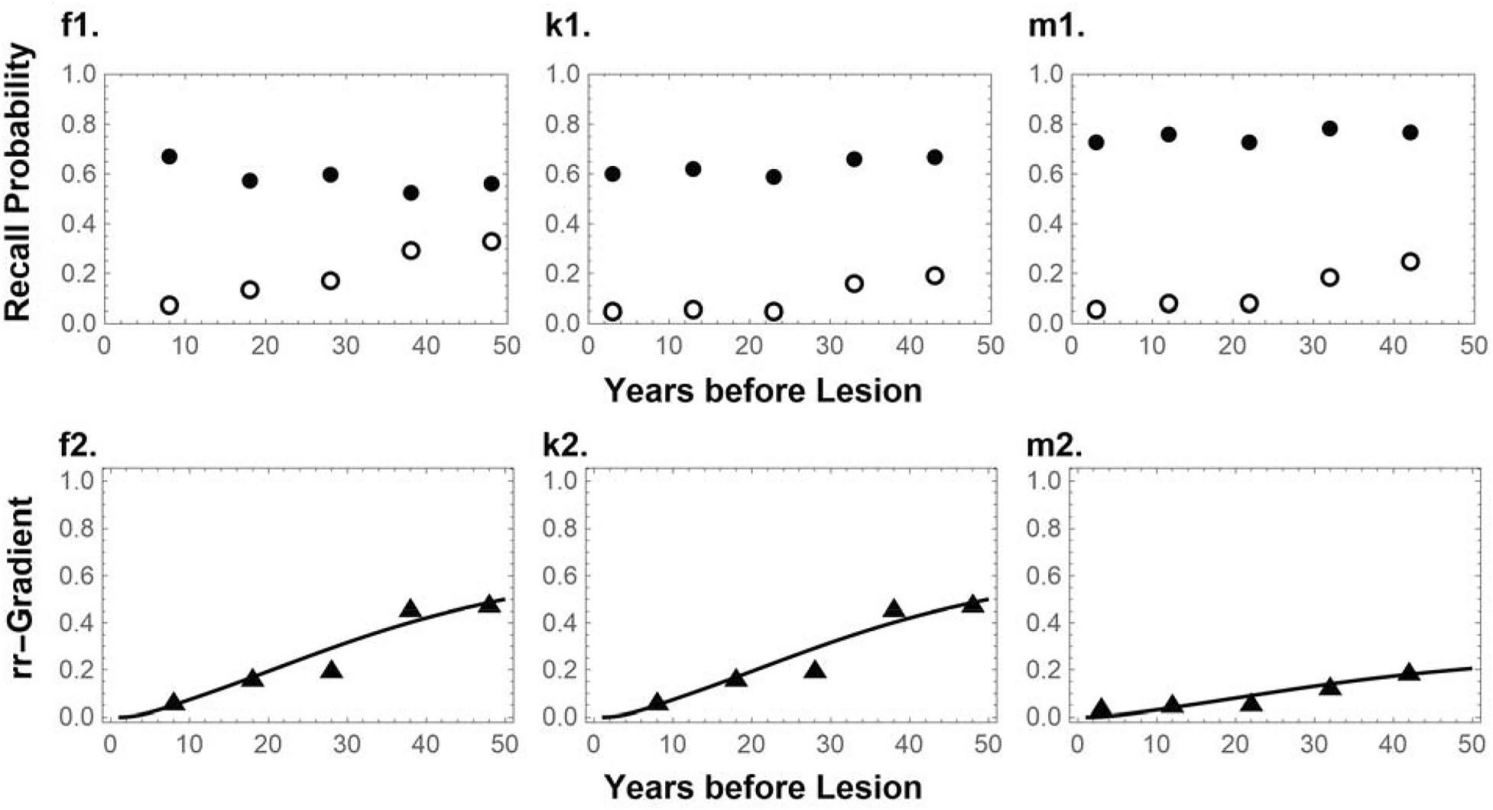
Questions about public events (**f1**), and famous faces and public events (**k1** and **m1**) spanning five decades answered by healthy controls (closed circles) and patients with Alzheimer's Dementia (open circles). The model (line) in **f2**, **k2**, and **m2** is fitted to the relative retrograde gradient, which is the ratio of the log-transformed probabilities (shown with triangles; see ^41^ for a background on the relative retrograde gradient). Data are taken from ^51^ for **f,** and from ^52^ for **k** and **m**. See the Supplementary Materials for a detailed description of the fitting procedure.

We fitted the model to three long-term forgetting curves and Ribot gradients of patients with Alzheimer’s Dementia, covering nearly half a century (see Fig. 2) illustrating that the Spine Drift Theory can in principle explain long-term Ribot gradients without recourse to a very long-lasting hippocampus-cortex dialog or multiple-trace replication mechanism (fits to ten additional data sets and full fitting details are available at https://osf.io/g5mqp/). It should be pointed out that the mechanism proposed here is not seen as a competing theory for either the ‘Standard Theory’, ‘Multiple Trace Theory’, or ‘Semantization Theory’^42^. Indeed, there is ample evidence that lesions of the hippocampus and surrounding areas can cause long-term Ribot gradients. For example, well-studied patients H.M and E.P. had such lesions and also were found to have retrograde amnesia gradients spanning 11 and 40-to-50 years, respectively^50^. Importantly, in these patients there was little evidence of wide-spread diffuse damage to the cortex, as found with Alzheimer’s Dementia.

If we assume that the hippocampus-cortex dialog plays an important role in consolidation (e.g., based on neural replay), then, according to the Spine Drift Theory proposed here, this dialog would still be subject to the neural mechanisms and perturbations observed. The only difference would be a preponderance of (relatively) recent memories being dependent on the hippocampal area compared with older memories. However, both areas would still show the behavior outlined above with diffuse lesioning. Hence both focal hippocampal lesions and diffuse hippocampal and cortical lesions would be able to cause long-term Ribot gradients. This would also form an alternative mechanism for Multiple-Trace Theory’s assumption of replication of traces: Instead of replicating traces, through reactivation, the synapses in older traces could reach more and more higher and stabler states with larger spines.

The theory also offers a possible neural basis for Jost’s Laws of Forgetting and Learning from 1897 (p.472, translated and rephrased)^8^, which state that that if two memory traces are of equal strength but different ages, the older one will (a) decay slower than the younger one and will (b) benefit more from additional learning. An in-depth review^53^ confirms that this is indeed a fair description of a large body of experimental data in memory psychology, but currently lacks grounding in neurobiology.

The theory introduced here surmises that a recently formed memory will have many connections in state 2, which is vulnerable to fluctuations to state 1 (i.e., the connection disappears). Hence, (a) it will decay faster than an old memory which has relatively more connections in higher, less plastic states. Furthermore, (b) a recent memory will have relatively few connections in state 1 (zero strength, Fig. 1b), which here is the only state affected by learning because state-1 connections can move to state-2 where they may become stabilized through activity-dependent plasticity (assumption *v*). Therefore, recent memories will benefit less than older memories from learning. For similar reasons, memories will have a lower forgetting rate when they have been learned in a spaced, rather than massed manner, because relatively many connections will have had the time to reach higher states due to the random state transitions and these are strong connections that decay more slowly. Equally strong memories learned in a massed manner rely upon large quantities of weaker memories that decay faster. Intrinsic fluctuations continue during the mere passing of time, thus, giving rise to an advantage of spaced over massed learning.

Concluding, the theory proposed here suggests a purpose for the paradoxically random movement of neural connection strengths in that it may implement a consolidation mechanism that slows down forgetting of older memories and safeguards them against diffuse lesioning by driving older memory traces to rely on smaller numbers but stronger connections, while at the same time freeing a large percentage of connections for new learning. As a side effect of this mechanism, classic memory laws of learning, forgetting, and amnesia emerge.

## Methods

In order to substantiate the conclusions drawn from the assumptions with the model, we must show the following:

1. We can select state transition probabilities ${p}_{ij}$ such that the equilibrium distribution resembles empirical distributions of connection strengths, while also have, C.2, transition probabilities for the highest states ${p}_{S,S-1}, {p}_{S-1,S-2},...,$ and ${p}_{S-1,S},{p}_{S-2,S-1},...,$ that are very low such that strong connections are not very plastic.
2. Transition probabilities can be set as in C.1 while allowing a sufficiently slow convergence to the equilibrium distribution (i.e., consolidation), possibly over many years.
3. Learning follows an exponential distribution.
4. Forgetting follows a power distribution.

Below, we will discuss each of these assumptions and analyze whether or not they are met by the proposed model. A Mathematica file (and its PDF) with the derivations and example plots is available in a repository at https://osf.io/g5mqp/ as a service to reader who wishes to pursue the derivations below in more depth.

### C.1 Equilibrium distribution

The assumptions of the theory are here developed with a Markov model in which each individual ‘connection’ conforms to a random walk with reflecting boundaries on *S* states, numbered 1 (zero strength) to *S* (highest strength). We prefer this approach to a continuous model^3^, because it gives more control over the shape of the equilibrium distribution and time parameters. At each point in (discrete) time, there is a non-zero probability ${p}_{ij}$ that a connection (synapse, spine) moves from its current state *i* to an adjacent higher or lower state *j* as given by the transition probability matrix *P*:

1 $$P=\left(\begin{array}{ccccccc}1-{x}_{2}{y}_{1}& {x}_{2}{y}_{1}& 0& 0& 0& \dots & 0\\ {x}_{1}{y}_{1}& 1-{x}_{1}{y}_{1}-{x}_{3}{y}_{2}& {x}_{3}{y}_{2}& 0& 0& \dots & 0\\ 0& {x}_{2}{y}_{2}& 1-{x}_{2}{y}_{2}-{x}_{4}{y}_{3}& {x}_{4}{y}_{3}& 0& \dots & 0\\ 0& 0& {x}_{3}{y}_{3}& 1-{x}_{3}{y}_{3}-{x}_{5}{y}_{4}& {x}_{5}{y}_{4}& \dots & 0\\ \dots & \dots & \dots & \dots & \dots & \ddots & \dots \\ 0& 0& 0& 0& 0& {y}_{S-1}{x}_{S-1}& 1-{y}_{S-1}{x}_{S-1}\end{array}\right)$$

In this tridiagonal matrix, rows add up to 1. It can easily be verified that $x=({x}_{1},{x}_{2},...,{x}_{S})$ is the equilibrium distribution by calculating $xP\text{, which gives }x,$ while the ${y}_{j}$ can be freely chosen. This allows us to simultaneously fit the model to an observed spine strength (equilibrium) distribution and—independently from this—to a certain time course of consolidation and forgetting.

### C.2 State life times

If we take the position of an ideal observer and follow a large population of neural connections, on average there will be a fraction of ${x}_{1}$ connections in the lowest state (zero or non-existent connection), ${x}_{2}$ in the smallest effective state, etc., and ${x}_{S}$ in the highest state (which contains large spines with low plasticity that cannot grow any stronger or larger). Intuitively, if we would observe a particular connection in state 2, there would be a relative high probability that it moves to state 3 (i.e., become stronger) or to state 1 (i.e., disappears). For a connection in (the highest) state *S*, however, the probability of moving to state *S*-1 is very low because we will chose a very low value for ${y}_{S-1}$. Hence, connections in high states (i.e., strong and large connections) represent very long-term memories.

More formally, we can calculate the estimated lifetimes as $[1-diag(P){]}^{-1}$, which for the highest state, for example, gives $({y}_{S-1}{x}_{S-1}{)}^{-1}$. If we set the time units for forgetting to days, we will require that the highest state may retain memories for over 25 years or, say, 10,000 days. If we set, for example, ${x}_{S-1}$ to 0.02 and ${y}_{S-1}$ to 0.005, then there is a 1/10,000 probability that a connection in the highest state spontaneously reverts to a lower one and an expected lifetime of 10,000 days. Dropping down one state does not imply forgetting of an entire memory, however, as we must take into account the strength contribution of each state of each connection in a group-to-group mapping formed in a learning event. We will, therefore, consider learning and forgetting in more detail.

### Learning

In order to show how the random walk model retains memories over time, we first define learning as forming a new mapping (input–output association) between *A* input and *B* output neurons, where each input neuron can in principle be connected to each output neuron (Fig. 1b). Learning itself is implemented by a learning rule, where a random fraction ${p}_{i,i+1}{\mu }_{i}$ of the input connections to each output neuron will move from state *i* to *i* + 1 per unit of learning time (one time unit is the default). In the simplified case we will consider here:

2 $${\mu }_{i}=\left\{\begin{array}{c} \mu , i=1\\ 0,i>1\end{array}\right.$$

In other words, learning stabilizes non-existing spines (in state 1) after they have transitioned into weak ones (in state 2) and it does not directly affect connections in higher states. (This limitation can easily be removed but this will complicate the model.) Each output neuron may receive up to *A* input connections; if there are no prior connections ('empty brain'), learning will cause a mean number of ${p}_{\mathrm{1,2}}\mu A$ connections to appear. We will generally assume, however, that the model is already at equilibrium, in which case only ${x}_{1}{p}_{\mathrm{1,2}}\mu A$ connections will be formed on average.

### Connection and input strength

Learning a mapping in the 'empty brain' results in each output neuron receiving on average ${p}_{\mathrm{1,2}}\mu A$ connections. To derive the strength of the net input to each output neuron, we assume that activations have values 0 (not activated) or 1 (activated) and that all *A* neurons in the input set are activated. We define the strength or *weight* of each state, denoted as $w(i)$, as proportional to its state number: $w(i)=\alpha \left(i-1\right)/\left(S-1\right)$ for states *i* from 1 to $S$, where $\alpha$ is a constant that is dependent on the type of measure. For simplicity we will here set $\alpha =1$ here and use $w(i)=\left(i-1\right)/\left(S-1\right)$. Note, however, that is quite easy to assign a different weight to each state without altering either the equilibrium distribution or the estimated state lifetimes. Also, there is no need to have the weights (as opposed to probabilities) add up to 1, unless one wants to introduce some type of normalization.

### C.3 Consolidation and forgetting

Can the time-course of consolidation and forgetting stretch over many years (in humans)? To analyze this, we define the fundamental matrix of the process as $Z=(I-P-X{)}^{-1}$, where *I* is the identity matrix and *X* the matrix consisting of identical row vectors *x*. A well-known result gives the first passage times as ${t}_{ij}=({z}_{jj}-{z}_{ij}){x}_{j}^{-1}$. In the model, we are particularly interested in ${t}_{2,S}$, which gives an estimate for the average time it takes for a newly formed connection (in state 2) to be fully consolidated to state *S*, keeping in mind that only a small fraction of the connections may reach this state during the lifetime of the process and that connections directly below the highest state will also contribute to resilience to forgetting and diffuse lesioning.

### Feedforward inhibition and retrieval

In the remainder, we will assume ‘learning at equilibrium’, so that each output neuron *b* will receive an expected increase in net input due to learning of $\Delta {\widehat{net}}_{b}={x}_{1}{p}_{\mathrm{1,2}}\mu A/(S-1)$. This is added to the expected net input at equilibrium, which is ${\widehat{net}}_{b}=A\sum w(i){x}_{i}=A(S-1{)}^{-1}\sum (i-1){x}_{i}$. This is the average net input to each output neuron *b* when activating the input pattern, but before any learning has taken place. To prevent output neurons from unwanted firing, we introduce feedforward inhibition ${F}_{b}$, which increases with the total number of activated input neurons *A*:

3 $${F}_{b}={\widehat{net}}_{b}+\frac{1}{2}\Delta {\widehat{net}}_{b}=A(S-1{)}^{-1}\left(\frac{{x}_{1}{p}_{\mathrm{1,2}}\mu }{2}+\sum (i-1){x}_{i}\right)$$

This implies that the signal due to learning arriving at neuron *b* from *A* input neurons is expected to be about $\frac{1}{2}\Delta {\widehat{net}}_{b}$.

(Alternatively, we observe that the equilibrium probability ${x}_{j}$ of state *j* has asymptotic variance ${\sigma }_{j}^{2}=2{x}_{j}{z}_{jj}-{x}_{j}-{x}_{j}^{2}$. We can then set the feedforward inhibition to the expected net input plus two times the weighed standard deviation, which would give an error signal of about 5%).

If we now introduce the following activation rule, we are able to later retrieve output pattern when the input pattern is presented, while filtering out background noise:

4 $$ac{t}_{b}=\left\{\begin{array}{ll}1, &\quad  {\text{if}} \, ne{t}_{b}-{F}_{b}>0\\ 0, &\quad {\text{otherwise}}\end{array}\right.$$

where $ac{t}_{b}$ is the activation value of output neuron *b*.

Due to chance fluctuations or prior learning of overlapping patterns, after activating a new input pattern with *A* neurons but before learning, a small fraction *f* of the output neurons may become activated (i.e., have $ac{t}_{b}=1$). After learning, however, when presenting the original input pattern most or all output neurons should be activated, depending on the learning rate parameter $\mu .$

### C.4 Learning has an exponential learning curve

Massed or continuous learning will move a constant fraction $f=\mu {x}_{2}{y}_{1},0<f<1,$ of neural connections from state 1 to state 2 until state 1 has been depleted. Starting with ${x}_{1}A$ connections in state 1 and ${x}_{2}A$ in state 2, at equilibrium, the expected number of connections after learning for *t* time units (learning time units are determined by μ), the remaining number of units in state 1 is ${x}_{t}A{\left(1-f\right)}^{t}$ and for state 2 we have $A{x}_{2}+A{x}_{1}-{x}_{t}A{\left(1-f\right)}^{t}.$ This is an exponentially decelerating learning curve with an asymptote at $A\left({x}_{1}+{x}_{2}\right)$.

### C.5 The shape of forgetting approximately conforms to a power forgetting curve in the recent portion and to an exponential curve in the tail

To analyze this, we will assume that $v$ represents the distribution of connection states just after having learned a new pattern (by having stabilized a certain number of connections that moved from state 1 to 2 in $x$, as described above). It is well-known that starting from any initial distribution $v$, we will with increasing time $t$ eventually reach the equilibrium distribution $x$, because $v{P}^{\infty }=x$. Once $x$ has been restored, we say that complete forgetting of $v$ has occurred. Of interest here is the shape and rate of this forgetting process. It can be shown that the shape of the forgetting process in a random walk process as studied here is dominated for high $t$ by the second eigenvalue of $P,$ ${\lambda }_{2}$. The convergence rate of the state distribution to the equilibrium distribution $x$ is of the shape ${a}_{2}{\lambda }_{2}^{-t}$ for some constant ${a}_{2}$^54^. This assumes that the eigenvalues are sorted from high to low. The lower eigenvalues also contribute to the shape of convergence—and hence forgetting—giving rise to a mixture of exponentials in the recent part of the forgetting curve; as more and more of these become very close to zero, the tail end of the curve approaches an exponential curve. Elsewhere, we have proven that mixtures of exponential curves under a fairly wide range of rate distributions tend to give rise to power functions in the limit^55^, which is a contributing factor to the ubiquitous nature of power functions in learning and memory, often called ‘Power Laws’.

A few more remarks must be made concerning C.5. (1) A detailed analysis of the expression for the second eigenvalue indicates that it in turn is dominated by the (low) plasticity of the highest state, which is determined by ${y}_{S}$. (2) It is almost never possible to derive simple closed-form solutions for the forgetting curves because the interacting transition probabilities are usually very hard to untangle. Once suitable values for all ${x}_{i}$ and ${y}_{j}$ in $P$ have been selected, however, the exact shape of forgetting can easily be calculated numerically and plotted. Computations confirm the assertions above about the initial and remote portions of the curves tending towards a power function and exponential function, respectively (examples are presented in a repository at https://osf.io/g5mqp/). (3) If the value for the highest state plasticity, ${y}_{S}$, is chosen very low, the forgetting curve may approach an asymptote: forgetting is not complete but reaches a non-zero plateau, which is in accordance with a large body of studies on forgetting in human long-term memory (so called *permastore*, e.g.,^56^). (4) Human and animal memory can be measured in countless ways, each introducing many factors that profoundly affect the expected shape of forgetting (savings, cued and free recall, recognition, choice, freezing and other postures, reaction times, etc.). Here, we have merely shown that the proposed theoretical processes of learning (C.4) and forgetting (C.5) conform to frequently observed curves and are as such not at odds with the data.

## Data Availability

All datasets analyzed during the current study are available in an Open Science Foundation repository at https://osf.io/g5mqp/. The repository also contains derivations and examples of the model in a Mathematical file (a PDF version is available as well).
